# Renal Failure and Systolic Heart Failure Have Synergistic Effect on In-Hospital All-Cause Mortality in Patients with Normotensive Acute Pulmonary Embolism

**DOI:** 10.3390/medsci13030183

**Published:** 2025-09-10

**Authors:** Mirjana Mijuskovic, Brankica Terzic, Sonja Salinger, Jovan Matijasevic, Sandra Pekovic, Tamara Preradovic-Kovacevic, Ljiljana Kos, Bjanka Bozovic, Irena Mitevska, Bojan Mitrovic, Aleksandar Neskovic, Ema Jevtic, Vladimir Miloradovic, Boris Dzudovic, Slobodan Obradovic

**Affiliations:** 1 Clinic of Nefrology, Military Medical Academy, School of Medicine University of Defense Belgrade, Serbia, 17 Crnotravska Street, 11040 Belgrade, Serbia; 2 Clinic of Cardiology, Clinical Center Nis, School of Medicine University of Nis, 18000 Nis, Serbia; 3 Institute for Pulmonary Diseases Vojvodina, School of Medicine University of Novi Sad, 21000 Novi Sad, Serbia; 4 Clinic of Cardiology, Clinical Center Banja Luka, School of Medicine University of Banja Luka, 78000 Banja Luka, Bosnia and Herzegovina; 5 Clinic of Cardiology, Clinical Center Montenegro, 81101 Podgorica, Montenegro; 6 Clinic of Cardiology, Clinical Center Skopje, School of Medicine University of Skopje, 1000 Skopje, North Macedonia; 7 Clinic of Cardiology, Clinical Center Zemun, School of Medicine University of Belgrade, 11040 Belgrade, Serbianeskovic@hotmail.com (A.N.); 8 Clinic of Cardiology, Clinical Center Kragujevac, School of Medicine University of Kragujevac, 34000 Kragujevac, Serbia; ema.jevtic@gmail.com (E.J.);; 9 Clinic of Emergency Internal Medicine, Military Medical Academy, School of Medicine University of Defense Belgrade, 11040 Belgrade, Serbia; 10 Clinic of Cardiology, Military Medical Academy, School of Medicine University of Defense Belgrade, Serbia, 11040 Belgrade, Serbia; sloba.d.obradovic@gmail.com

**Keywords:** pulmonary embolism, systolic heart failure, renal failure

## Abstract

**Background/Objectives**: Renal failure (RF) and systolic heart failure (sHF) are very often associated with each other, and their synergistic influence can affect the prognosis of acute pulmonary embolism (aPE) patients. The aim of this study is to evaluate the associations between RF, sHF, and in-hospital mortality in patients with normotensive aPE. **Methods**: We analyzed data from the Regional PE Registry (REPER), and 1968 patients with CT pulmonary angiography-confirmed aPE who had a systolic blood pressure of 100 mmHg and higher, and for whom creatinine blood levels and left ventricular ejection fraction (LVEF) were measured at admission to hospital were enrolled. The patients were divided into four groups: the first group comprised patients without renal and systolic heart failure, the second those with RF (creatinine clearance less than 60 mL/min), the third those with sHF (LVEF less than 50%), and the fourth those with both RF and sHF. The primary endpoint of this study was in-hospital all-cause mortality. **Results**: There are significant differences between in-hospital mortality among the groups: 38/1247 (3.0%) vs. 63/514 (12.9%) vs. 10/99 (10.1%) vs. 20/108 (18.5%) (*p* < 0.001). In the multivariable regression model adjusted for age, right ventricular dysfunction, and troponin levels, the presence of renal failure, sHF, and both were independently associated with in-hospital all-cause mortality with ORs of 3.59 (95%CI 2.04–6.30, *p* < 0.001) vs. 3.97 (1.71–9.25, *p* = 0.001) vs. 6.39 (3.15–12.99, *p* < 0.001), respectively. **Conclusions**: The association of renal failure and systolic heart failure has a deleterious prognosis in patients with normotensive aPE.

## 1. Introduction

Patients with heart failure (HF) and renal failure (RF) share multiple pathophysiological mechanisms that promote a hypercoagulable state and predispose them to pulmonary embolism (PE) [1,2]. Both conditions are characterized by endothelial dysfunction, reflected in reduced nitric oxide (NO) secretion and increased von Willebrand factor release, elevated levels of proinflammatory cytokines, enhanced platelet aggregation, and impaired fibrinolysis due to increased plasminogen activator inhibitor-1 (PAI-1) concentrations [3,4]. These alterations, often accompanied by reduced mobility, contribute to a prothrombotic milieu.

Heart failure is recognized as an important independent predictor of PE, with risk increasing as left ventricular systolic function deteriorates [5]. Similarly, renal impairment has been consistently associated with a higher risk of PE, which rises progressively with declining kidney function [6,7]. Importantly, both HF and RF independently contribute to increased mortality in patients with acute PE (aPE).

Epidemiological data indicate that left ventricular systolic dysfunction is present in 6.2–12% of patients with aPE and is associated with higher rates of adverse outcomes, including mortality [5,8,9,10]. Accordingly, HF has been incorporated into most validated PE mortality prediction models [11]. In contrast, although chronic kidney disease (CKD) and end-stage renal disease (ESRD) are established risk factors for PE incidence and prognosis, impaired renal function has not yet been integrated into existing prediction models such as the sPESI score. Data from large cohorts have shown that patients with CKD and ESRD have markedly higher incidence rates of PE, worse in-hospital outcomes, and significantly increased risk of both recurrent venous thromboembolism and major bleeding [12,13,14]. Furthermore, acute kidney injury (AKI) is frequent in normotensive PE, occurring in about 20% of patients, and is strongly associated with right ventricular dysfunction and pre-existing CKD [15].

Despite robust evidence highlighting the prognostic significance of renal impairment in aPE, its role in risk stratification remains underappreciated. Given the well-recognized bidirectional interaction between the heart and kidneys, the synergistic effect of HF and RF may have important implications for outcomes in patients with aPE [16,17].

The aim of this study is to evaluate the associations between RF, sHF, and in-hospital mortality in patients with normotensive aPE.

## 2. Materials and Methods

The data for this investigation were sourced from the Regional PE Registry (REPER), which is a multicenter, multinational registry of hospitalized PE patients with CTPA-confirmed aPE. Nine hospitals from 4 western Balkan countries have joined the REPER. The Military Medical Academy from Belgrade is the founder of the REPER, and the collection of data started in 2015. Clinical University Center Zemun, Institute of Pulmonary Diseases Vojvodine from Sremska Kamenica, University Clinical Center Nis, University Clinical Center Kragujevac, and General Hospital Pancevo are the hospitals based in Serbia. University Clinical Center Banja Luka in Bosnia and Herzegovina, University Clinical Center Podgorica in Montenegro, and University Clinical Center Skopje in North Macedonia are also members of the registry. All centers’ Review Boards or Ethical Committees gave permission for the data collection and publishing of the results from the REPER without using personal data. This study was approved by the institutional ethics committees of all participating centers (Decision of the Ethics Committee of the central research institution, the Military Medical Academy, No. 98/2023, 3 January 2024). All investigations were conducted in accordance with the principles of the Declaration of Helsinki [18]. In most participating institutions, registry data are managed by a senior physician—usually the head of department—who oversees patient care and data quality, and a junior doctor, who ensures accurate data entry and submits updates to the central administrator. The patients were informed that the data on their disease were collected in the regional database without revealing their identity and personal data.

Arterial blood pressure in the REPER database was measured upon hospital admission in the ward where patients were subsequently hospitalized. Since initial fluid resuscitation and oxygen therapy administered in the ambulance or emergency department could have influenced hemodynamic status, only measurements obtained in the hospital ward were considered for analysis.

**Figure 1:**
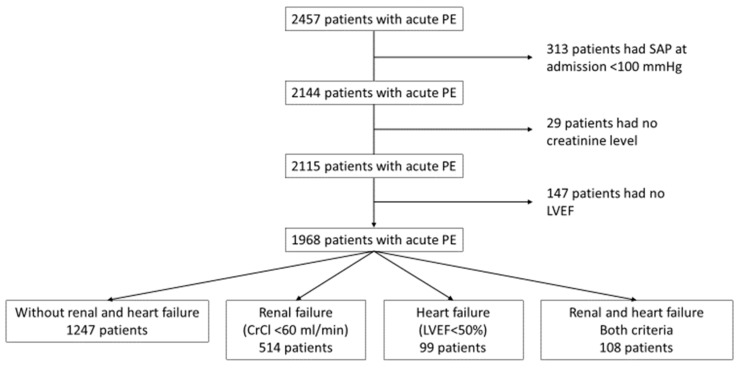
Flow chart of this study.

According to the REPER protocol, we tried to ensure that all patients had a similar diagnostic algorithm and creatinine blood levels were available for almost all patients. Transthoracic examination at admission is also recommended, but not all institutions had 24/7 accessibility to echocardiography and 7% of patients did not undergo this examination at admission (a flow chart of this study is presented in Figure 1).

The main purpose of transthoracic echocardiography was to identify RV dysfunction by measuring RV diameters, tricuspid regurgitation velocity and RV systolic pressure, McConnell sign, acceleration time in the RV outflow tract, and tricuspid annular plane systolic excursion (TAPSE) [19]. However, almost all kinds of echocardiography also provide data about LV ejection fraction, measured with various methods at the discretion of the attending echocardiographer. Patients were divided into two groups according to LVEF: patients without systolic heart failure with LVEF ≥ 50%, and the group of patients with systolic heart failure and LVEF < 50%. The majority of patients with systolic heart failure had at least one previous echocardiography imaging which confirmed the diagnosis of LVEF systolic dysfunction.

According to creatinine blood levels, sex, body mass, and age, we calculated creatinine clearance using the Cockcroft–Gault formula and MDCalc (an online calculator) [20]. Patients with CrCl < 60 mL/min were considered as patients with renal failure. We tried to avoid the influence of hypotension on CrCl and excluded patients with systolic arterial pressure of less than 100 mmHg at admission from our investigation. We also calculated the estimated glomerular filtration rate (eGFR) using the CKD-EPI equation [21], and the main results with this calculation are presented in Supplementary Table S1 and Supplementary Figure S1.

Right ventricular dysfunction was defined as the presence of a CTPA indication of an increased RV/LV diameter ratio greater than 1 at the four-chamber view just below the tricuspid and mitral anulus, or using echocardiography parameters (the presence of at least two parameters of four, dilated RV diameter greater than 4 cm just below the tricuspid annulus, TAPSE < 1.6 cm, RVSP > 40 mmHg, or the presence of McConnell sign) [19].

Cardiac troponin and BNP or NT-proBNP blood concentrations were measured at admission according to the standard procedures of the different institutions using central biochemical laboratories, not point-of-care methods. Trained physicians recorded all necessary patient data according to the predefined REPER protocol. The primary endpoint of this study was in-hospital all-cause death. Major bleeding was recording during hospitalization using International Society on Thrombosis and Haemostasis (ISTH) criteria [22].

### Statistics

Patients with normotensive acute PE with measured creatinine blood levels and known LVEF were divided into four groups (Figure 1): those without renal and systolic heart failure, those with renal failure, those with systolic heart failure, or those with both. The main characteristics of patients are presented as frequencies and the significance of differences between these characteristics was estimated using the Hi-square test (Table 1). The differences in age were estimated using a one-way ANOVA test. The main risk factors for in-hospital all-cause death were analyzed with univariable and multivariable binary regression analysis. Odds ratios, 95% confidence intervals, and *p* values were calculated. Two multivariable regression models were used to determine which variables are independently associated with all-cause death. In the first model, only demographic parameters that are available for all patients were used. In the second model, we used only patients with determined RV dysfunction and had measured cTn blood level at admission (Table 2). Multicollinearity statistics were obtained using linear regression models with the same parameters as in the two binary regression models, and variance inflation factors (VIFs) for each variable in the model are presented in Table 3. *p* values of less than 0.05 are considered significant.

The statistical analysis of the data was performed using the software IBM SPSS 26.0.

## 3. Results

### 3.1. Demographic Characteristics and Comorbidities

The mean age of the study population was 59 years (SD 16), and 53.5% were female. Among the 2457 patients with acute PE enrolled in the REPER registry, 313 had systolic arterial blood pressure at admission of less than 100 mmHg, 29 had no measured creatinine blood levels at admission, and 147 had no estimation of LVEF at admission. These patients are excluded from this analysis (a flow chart of this study is presented in Figure 1). Therefore, 1968 patients with acute PE were divided into four groups: the first group with 1247 (63.4%) patients had no renal and heart failure, the second group had 514 (26.1%) patients with renal failure, the third group had 99 (5.0%) patients with heart failure, and the fourth group consisted of 108 (5.5%) patients who had both renal and heart failure.

The basic characteristics of the patients according to the presence of renal and heart failure are presented in Table 1.

The highest incidence of pulmonary embolism (PE) among patients under 60 years of age was observed in the heart failure group (34.3% vs. 9.3% in the renal failure group, vs. 4.6% in the combined renal and heart failure group). In contrast, among patients older than 75 years, the highest incidence of PE was noted in the group with combined renal and heart failure (56.5%) and the renal failure group (50.2%), compared to the remaining two groups (Table 1). Analysis of comorbidities revealed that the prevalence of chronic obstructive pulmonary disease (COPD), coronary artery disease (CAD), arterial hypertension, diabetes mellitus, atrial fibrillation, and previous stroke was significantly higher in patients with both renal and heart failure compared to the other groups (*p* < 0.001) (Table 1). A statistically significant difference was also observed in the prevalence of major bleeding within 21 days prior to PE (*p* = 0.002). No significant differences were found among the groups in the prevalence of anemia, active malignancy, or history of previous bleeding (Table 1). A significantly higher proportion of patients with GFR < 30 mL/min/1.73 m^2^ was present in the combined renal and heart failure group (23.1%) compared to the renal failure group (18.3%). There were no patients with a GFR lower than 30 mL/min/1.73 m2 in the group with heart failure and the group of patients who had neither renal nor heart failure. None of the patients in the study cohort were maintained on dialysis.

### 3.2. Basic Clinical, Laboratory, and Imaging Parameters at Admission

#### 3.2.1. Clinical Parameters

There were statistically significant differences among the groups regarding the presence of hemoptysis (*p* < 0.001), chest pain (*p* < 0.001), and heart rate > 110 bpm (*p* < 0.002), with the highest frequency of these symptoms found in the heart failure group (Table 2). No significant differences were observed between the groups in terms of prevalence of dyspnea, syncope, and fever (Table 2).

#### 3.2.2. Laboratory Parameters

The levels of brain natriuretic peptide (BNP) and cardiac troponin T (cTnT) differed significantly among the groups (*p* < 0.001). BNP > 100 ng/mL was present at a similar frequency in the heart failure and combined renal and heart failure groups (86.9% vs. 86.4%) and was significantly more frequent than in the other two groups. Elevated cTnT level was most prevalent in the combined renal and heart failure group (72%) (Table 2).

#### 3.2.3. Imaging Parameters

Right ventricular dysfunction showed a statistically significant difference among the groups (*p* < 0.001), with the highest frequency in patients with combined renal and heart failure (76.8%) (Table 2).

### 3.3. Treatment of Patients

Anticoagulant therapy at admission and during the first days after hospitalization was differently distributed throughout the four subgroups, and these data are presented in Table S2. Between 30 and 35% of patients without renal and systolic heart failure and with renal failure more often only received unfractionated heparin as the first-line anticoagulation, compared to 18–20% of patients with only heart and combined renal and heart failure. By contrast, low molecular weight heparin was used as a predominant anticoagulation in only 72–76% of heart failure and combined renal and heart failure patients, compared to 62–66% of patients without renal and heart failure and only renal failure.

A reperfusion strategy was applied according to the discretion of the attending physicians, and it was not significantly different among the groups (Table S3). Classic full-dose tPA systemic intravenous thrombolysis was given in about 15–17% of patients without renal and heart failure and in the only renal failure group, whereas it was implemented in 8–11% of patients in the other two groups. Catheter-directed thrombolysis with a dose of tPA of less than 30 mg was given to a similar number of patients in the four groups (between 2.9 and 5.1%). Reperfusion therapy was given if patients deteriorated hemodynamically or if there was progression of some vital parameters which indicated the worsening of the borderline clinical state, mostly in intermediate/high-risk patients (increase in respiratory rate, decrease in oxygen saturation, increase in heart frequency, or drop in systolic blood pressure).

### 3.4. Overall Mortality and Major Bleeding

**Figure 2:**
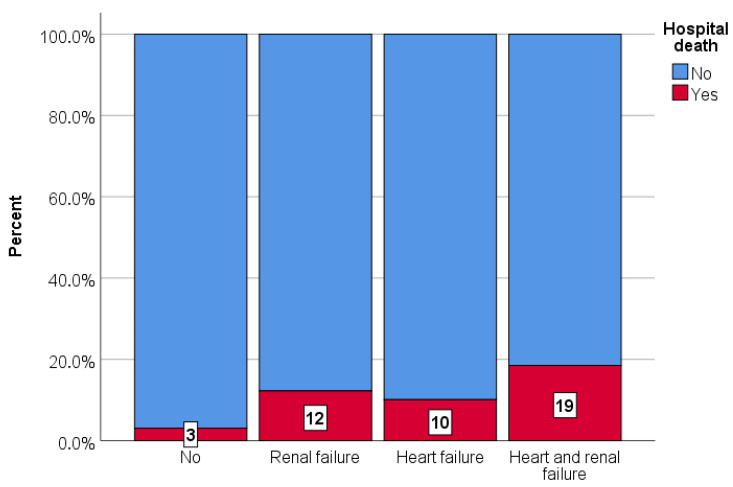
In-hospital all-cause death of acute, normotensive PE patients regarding the presence of renal failure, heart failure, or both (*p* < 0.001).

There are significant differences between hospital mortality among the groups: 38/1247 (3.0%) vs. 63/514 (12.9%) vs. 10/99 (10.1%) vs. 20/108 (18.5%) (*p* < 0.001) (Figure 2). Major bleeding according to ISTH criteria was similar between the groups (5.9% vs. 6.0%, vs. 1.0% vs. 5.6%, *p* = 0.231).

### 3.5. Univariable and Multivariable Regression Analysis

The univariable and multivariable regression analysis for the prediction of in-hospital mortality in normotensive patients with PE is detailed in Table 3.

In the univariable analysis, only variables which have significant independent association with in-hospital mortality are presented in Table 3: age, COPD, active cancer, diabetes, heart rate greater than 110 bpm at admission, RV dysfunction, and elevated cTn. Gender was not a significant predictor (Table 3).

In the multivariable regression model adjusted to age, right ventricular dysfunction, and troponin levels, the presence of renal failure, sHF, and both were independently associated with in-hospital all-cause mortality with ORs of 3.59 (95%CI 2.04–6.30, *p* < 0.001) vs. 3.97 (1.71–9.25, *p* = 0.001) vs. 6.39 (3.15–12.99, *p* < 0.001), respectively. Using linear regression models with the same variables as in binary regression models 1 and 2, we were able to test multicollinearity between variables. We found that all variance inflation factors (VIFs) are less than 3, which means there is no significant multicollinearity between the tested variables (Table 3).

## 4. Discussion

The coexistence of renal failure (RF) and heart failure (HF) significantly increases the risk of patients developing acute pulmonary embolism (aPE). In addition to this increased risk, both conditions are independent predictors of mortality in these patients. Early mortality rates in patients with HF and aPE vary between 1.3% and 4.2%, depending on the severity of PE among patients included in studies [23,24]. In patients with RF, early mortality rates are similar, ranging from 1.15% to 4.2% depending on the degree of renal dysfunction [25,26].

To our knowledge, the present study appears to be the first to assess the impact of the simultaneous presence of renal failure (RF) and systolic heart failure (sHF) on early mortality in patients with aPE. Significant differences were observed in in-hospital mortality across groups: 38/1247 (3.0%) in the group without RF and sHF, 63/514 (12.9%) in the RF group, 10/99 (10.1%) in the sHF group, and 20/108 (18.5%) in the group with both RF and sHF (*p* < 0.001). The presence of either RF or sHF alone was associated with an approximately threefold increase in mortality risk after adjustment for confounding factors, while their coexistence had a synergistic effect. When both conditions were present, the risk of mortality increased approximately sixfold—ORs of 3.59 (95% CI 2.04–6.30, *p* < 0.001), 3.97 (1.71–9.25, *p* = 0.001), and 6.39 (3.15–12.99, *p* < 0.001).

The prevalence of left ventricular systolic dysfunction in aPE ranges from 5.1% to 12% and represents one of the strongest predictors of early mortality [9,27]. In the study by Liteplo et al., which monitored various echocardiographic parameters, only LV systolic dysfunction was a statistically significant predictor of 30-day mortality (OR 9.63, 95% CI 1.74–53.32) [5]. The presence of HFrEF—but not HFpEF—was an independent predictor of 7-day mortality in patients with aPE [10]. In the study by Cires-Drouet et al., in-hospital mortality in patients with aPE and LV systolic dysfunction was 12.8% compared to 7.4% in those with normal LV function [8]. A similar mortality rate was observed in our sHF group (10.1%).

Studies on aPE and LV systolic dysfunction have shown that a significant proportion of these patients had no prior history of heart failure or coronary artery disease, were younger, and exhibited global hypokinesia, suggesting acute onset sHF [9,28]. The mechanism underlying acute LV dysfunction in aPE involves a higher incidence of massive PE in this subgroup, leading to sudden increases in pulmonary vascular resistance, right ventricular dilatation, and leftward septal shift, compromising LV preload [29,30]. Additionally, increased end-diastolic pressure may impair coronary perfusion, inducing myocardial ischemia, which together contribute to acute sHF. Therefore, the presence of sHF can exacerbate aPE (and vice versa), significantly affecting patient survival [28].

In our study population, the highest proportion of patients under 60 years of age (34.3%) was observed in the sHF group, suggesting that many of these patients may have had acute LV systolic dysfunction. In contrast, the highest prevalence of patients over 75 years old was found in the group with both RF and sHF (56.5%), suggesting a likely higher presence of chronic HF, which is more common in older individuals and those with RF, as confirmed by other authors [13,31].

Patients with RF often have multiple cardiovascular comorbidities [14]. A meta-analysis by Zhan et al. showed that the presence of chronic heart failure (CHF), coronary artery disease (CAD), and atrial fibrillation (AF) increases the risk of venous thromboembolism (VTE). Patients with RF and CAD had a 28% higher risk of VTE, and those with RF and AF had a 97% higher risk compared to patients without these comorbidities [6,32]. In our study, patients with both RF and sHF had significantly higher rates of comorbidities such as CAD, COPD, type 2 diabetes mellitus, and AF than the other three groups (Table 2). These comorbidities are known risk factors for both the development of aPE and higher mortality, as confirmed by our findings.

Plasma creatinine levels and estimated glomerular filtration rate (eGFR) are used to assess renal function. In our study, renal function, expressed by creatinine clearance, was initially assessed using the Cockcroft–Gault formula. However, it is known that this formula tends to overestimate the GFR due to its reliance on body weight. Therefore, the eGFR was also calculated using the CKD-EPI (Chronic Kidney Disease Epidemiology Collaboration) equation, which is currently considered the most accurate method for estimating kidney function [33]. The study by Altinsoy et al. demonstrated that estimating the GFR with the CKD-EPI equation may serve as a prognostic marker in normotensive patients with aPE [34]. Applying both formulas in our study did not yield significant differences in patient group distribution or mortality rates (Table S3 and Figure S1).

Acute PE is often accompanied by hypotension, which may adversely affect kidney function. However, even normotensive aPE patients can experience renal impairment [34]. The main predictors of GFR reduction in aPE include reduced renal arterial blood flow (RABF) due to decreased cardiac output (CO) and renal venous congestion [35,36]. Normal blood pressure in patients with aPE is maintained despite reduced CO due to increased neurohumoral activity, which involves elevated catecholamine release and sympathetic overactivity. Both HF and aPE are associated with such neurohumoral activation, and blood urea nitrogen (BUN) is one of the most significant biomarkers of this process. Studies have shown a correlation between BUN levels and survival in HF patients, while eGFR appears to be a better survival predictor in aPE patients [24,37].

In our group of patients with both RF and sHF, the highest proportion of patients with eGFR < 30 mL/min/1.73 m^2^ was observed: 25/108 (23.1%) (*p* < 0.001). LV systolic dysfunction had a significant impact on the hemodynamic status of this vulnerable patient population and contributed to the worsening of pre-existing renal impairment. An additional factor potentially influencing renal function deterioration in patients with RF or both RF and sHF was the use of contrast agents during CTPA. Moreover, HF is a known predictor of contrast-induced nephropathy, which underscores the need for caution. The presence of RF in acute PE is a marker of worse short- and long-term outcomes [7,24,26]. Previous studies suggest that including eGFR < 60 mL/min/1.73 m^2^ as an additional risk stratification criterion could improve the performance of risk scores such as the simplified PE severity index (sPESI) [38]. Based on our results, we emphasize the need to assess creatinine clearance and LVEF at admission in normotensive PE patients. Reduced CrCl (<60 or <30 mL/min) and LVEF < 50% are both associated with increased mortality risk, while their coexistence identifies patients with a prognosis comparable to high-risk PE. Such patients should be closely monitored and considered for reperfusion therapy.

Patients with renal insufficiency have an increased risk of both thrombosis and major bleeding compared to those without kidney dysfunction [15,39]. Additionally, anticoagulant therapy in these patients can be challenging due to reduced renal drug clearance. The GARFIELD-VTE (Global Anticoagulant Registry in the Field—Venous Thromboembolism) study showed that patients with moderate-to-severe RF had a higher risk of major bleeding than those with mild or no RF. Furthermore, patients with moderate-to-severe RF were less likely to receive direct oral anticoagulants [40]. In our study, the main differences in initial anticoagulant therapy were a more frequent use of nonfractional heparin (UFH) in patients without RF and HF, as well as in patients with RF only. One possible explanation is that these two groups were more often treated with thrombolysis, for which UFH is advantageous due to the ability to monitor APTT and the option to reverse its effect with protamine. Conversely, patients with HF may have been perceived as more complex cases, making the use of fixed-dose low molecular weight heparin (LMWH) more convenient for clinicians compared with the less predictable management of UFH. Our study showed that major bleeding, according to ISTH criteria, was similar between groups (5.9% vs. 6.0% vs. 1.0% vs. 5.6%, *p* = 0.231).

Right ventricular dilation in patients with acute pulmonary embolism (aPE) leads to increased release of pro–B-type natriuretic peptide (BNP), which is rapidly cleaved into BNP and N-terminal proBNP (NT-proBNP). BNP is considered one of the most important biomarkers of adverse outcomes in these patients. The half-life of BNP is shorter than that of NT-proBNP, and both are eliminated through the kidneys. There is a negative correlation between the concentrations of these biomarkers and renal function [41]. Meta-analyses have shown that patients with aPE and elevated levels of BNP or NT-proBNP have a six- to sevenfold increased risk of mortality [42]. In our cohort, among patients with systolic heart failure (sHF) and those with both sHF and renal failure (RF), the highest proportion had BNP > 100 ng/mL, which was associated with fatal outcomes.

Cardiac troponin (cTn) levels may also be elevated in patients with aPE and carry significant prognostic value. A large meta-analysis involving 1985 patients with PE demonstrated that elevated cTn levels, even among hemodynamically stable patients, were associated with an increased risk of mortality. It is well known that renal function influences cTn levels [43]. The Chronic Renal Insufficiency Cohort (CRIC) study showed that 81% of patients had elevated cTn values in the absence of cardiovascular disease, with a significant increase observed as the glomerular filtration rate (GFR) decreased [44]. In our study population, the highest proportion of patients with elevated cTn levels (72%) was observed in the group with concomitant sHF and RF, which was associated with poor prognosis.

Patients with aPE and sHF are less likely to receive advanced interventions, which was also the case in our cohort [45]. The lowest frequency of reperfusion therapy—including thrombolysis and interventional procedures—was observed in the groups with sHF and RF (13.8%) and sHF alone (17.2%). One possible explanation is that patients with HF were more frequently on anticoagulant or antiplatelet therapy, which are often considered relative contraindications for thrombolysis. Another important factor is that patients with HF exhibited a lower thrombus burden compared with the other three groups, as some clinicians consider CTPA-derived thrombus parameters when deciding on thrombolytic therapy.

## 5. Conclusions

Renal failure and systolic heart failure are frequently associated in patients with acute PE, and each condition individually has a negative impact on both short- and long-term survival in these patients. The association of renal failure and systolic heart failure results in a deleterious prognosis in patients with normotensive aPE.

## Figures and Tables

**Table 1:** Demographic characteristics of patients with PE.

	No.	Renal Failure	Heart Failure	Renal/Heart Failure	*p*
Age, y mean ± SD	59 ± 16	74 ± 11	64 ± 14	76 ± 9	<0.001
Female sex, *n* (%)	653 (52.4)	318 (61.9)	30 (30.3)	51 (47.2)	<0.001
<60 years, *n* (%)	543 (43.5)	48 (9.3)	34 (34.3)	5 (4.6)	<0.001
>75 years, *n* (%)	158 (12.7)	258 (50.2)	24 (24.2)	61 (56.5)	<0.001
Comorbidities					
COPD, *n* (%)	101 (8.1)	74 (14.4)	12 (12.1)	18 (16.7)	<0.001
Major surgery (21 days before PE) *n* (%)	224 (18)	59 (11.5)	10 (10.1)	20 (18.5)	0.002
Arterial hypertension, *n* (%)	702 (56.5)	393 (76.6)	66 (66.7)	77 (71.3)	<0.001
Coronary disease, *n* (%)	97 (7.8)	67 (13)	40 (40.4)	50 (46.3)	<0.001
DM type 2, *n* (%)	197 (15.8)	139 (27)	27 (27.3)	38 (35.2)	<0.001
Previous stroke, *n* (%)	57 (4.6)	52 (10.1)	8 (8.1)	17 (15.7)	<0.001
Drugs (antiplatelets, CS, NSAIL), *n* (%)	281 (22.6)	134 (26.3)	34 (34.3)	47 (43.5)	<0.001
Previous bleeding, *n* (%)	69 (5.6)	28 (5.4)	3 (3)	7 (6.5)	ns
Atrial fibrillation, *n* (%)	96 (7.8)	99 (19.8)	36 (37.5)	42 (42)	<0.001
Anemia, *n* (%)	290 (23.3)	137 (26.8)	26 (26.3)	31 (29.2)	ns
GFR < 30 mL/min/1.73 m^2^, *n* (%)	0(0)	94 (18.3)	0 (0)	25 (23.1)	<0.001
Active malignant disease, *n* (%)	154 (12.3)	69 (13.4)	12 (12.1)	9 (8.3)	ns
Time from the onset of symptoms to admission to hospital, hours; median (IQR)		72 (24–120)	96 (36–240)	72 (24–160)	0.02
Duration of hospital stay, days; median (IQR)		10 (7–14)	10 (7–14)	11 (8–14)	0.0286
CTPA thrombotic burden; median (IQR)		12 (8–18)	8 (5–13)	12 (7–15)	0.022

COPD—chronic obstructive pulmonary disease; CS—corticosteroids; NSAIL—nonsteroidal anti-inflammatory drugs; GFR—glomerular filtration rate.

**Table 2:** Basic clinical, laboratory, and imaging parameters at admission.

Symptoms	No.	Renal Failure	Heart Failure	Renal/Heart Failure	*p*
Dyspnea, *n* (%)	1014 (81.4)	446 (86.9)	86 (86.9)	94 (87)	ns
Hemoptysis, *n* (%)	100 (8)	14 (2.7)	9 (9.1)	8 (7.4)	<0.001
Syncope, *n* (%)	159 (12.8)	62 (12.1)	9 (9.1)	16 (15)	ns
Chest pain, *n* (%)	528 (42.4)	160 (31.2)	44 (44.4)	41 (38)	<0.001
Fever, *n* (%)	196 (15.7)	65 (12.7)	16 (16.2)	11 (10.2)	ns
Heart rate > 110/min, *n* (%)	331 (26.5)	139 (27)	43 (43.4)	36 (33.3)	<0.002
Dysfunction of RV, *n* (%)	666 (58.9)	329 (70.8)	67 (74.4)	73 (76.8)	<0.001
BNP > 100 pg/mL, *n* (%)	381 (49.9)	241 (75.1)	53 (86.9)	57 (86.4)	<0.001
cTnT (xUNRL) *n* (%)	484 (51.1)	258 (65.8)	45 (60.8)	67 (72)	<0.001
LVEF (<50%); median (IQR)		60 (55–65)	37 (25–43.5)	40 (30–45)	<0.001
GFR (mL/min/1.73 m^2^); median (IQR)		43.5 (32.7–52.4)	78 (67.1–96.3)	35.3 (25.3–47.7)	<0.001
ClCr (mL/min); median (IQR)		47 (37.4–55)	85 (68.3–104)	40.6 (31.5–57.6)	<0.001

RV—right ventricle; BNP—brain natriuretic peptide; cTnT—cardiac troponin T; UNRL—upper normal range limit; LVEF—left ventricular ejection fraction; GFR—glomerular filtration rate; ClCr—creatinine clearance.

**Table 3:** Univariable and multivariable regression analysis for the risk factors for in-hospital all-cause death. The first model uses only demographic data and heart rate at admission which are available in all patients. In the second group, patients who did not have determined RV dysfunction and/or cTn blood levels at admission are missing. The collinearity statistics are represented by variance inflation factor (VIF) values.

Variables	Univariable Binary Regression Odds Ratio (95% CI, *p*)	Model 1—Only Demographic Variables Multivariable Binary Regression Odds Ratio (95% CI, *p*, VIF)	Model 2—Demographic Variables with RV Dysfunction and cTn Blood Level Multivariable Binary Regression Odds Ratio (95% CI, *p*, VIF)
Age	1.04 (1.02–1.05, *p* < 0.001)	1.02 (1.00–1.03, *p* = 0.003, VIF = 1.18)	1.014 (0.99–1.03, *p* = 0.169, VIF = 1.18)
Female sex vs. male sex	1.22 (0.85–1.74, *p* = 0.279)	-	
COPD vs. no COPD	1.71 (1.05–2.80, *p* = 0.031)	-	-
Active cancer	1.84 (1.17–2.89, *p* = 0.008)	1.93 (1.21–3.10, *p* = 0.006, VIF = 1.01)	1.86 (1.03–3.35, *p* = 0.038, VIF = 1.004)
Diabetes mellitus	2.10 (1.43–3.07, *p* < 0.001)	1.57 (1.06–2.33, *p* = 0.026, VIF = 1.04)	-
Heart rate > 100 vs. ≤100 bpm	1.73 (1.12–2.47, *p* = 0.003)	1.73 (1.20–2.51, *p* = 0.003, VIF = 1.02)	-
Renal and/or systolic heart failure		(VIF = 1.17)	(VIF = 1.16)
No	1.00	1.00	1.00
Renal failure CCl < 60 mL/min	4.44 (2.93–6.47, *p* < 0.001)	3.30 (2.08–5.26, *p* < 0.001, VIF = 1.28)	3.59 (2.04–6.30, *p* < 0.001, VIF = 1.30)
Heart failure LVEF < 50%	3.57 (1.72–7.41, *p* = 0.001)	2.95 (1.41–6.19, *p* = 0.004, VIF = 1.04)	3.97 (1.71–9.25, *p* = 0.001, VIF = 1.04)
Both renal and heart failure	7.23 (4.04–1.96, *p* < 0.001)	5.07 (2.69–9.57, *p* < 0.001)	6.39 (3.15–12.99, *p* < 0.001)
RV dysfunction	1.79 (1.15–2.77, *p* > 0.001)	-	-
Elevated cTn at admission	3.30 (1.98–5.14, *p* < 0.001)	-	2.63 (1.56–4.47, *p* < 0.001, VIF = 1.06)

## Data Availability

The original contributions presented in this study are included in the article; further inquiries can be directed to the corresponding author.

## Supplementary Materials

The following supporting information can be downloaded at: https://www.mdpi.com/article/10.3390/medsci13030183/s1, Figure S1: All-cause hospital death of acute, normotensive PE patients regarding the presence of renal failure using eGFR, heart failure or both (p<0.001); Table S1: All-cause death according the eGFR definition of renal failure (<60 mL/min/1.73m^2^) in patients without renal and heart failure, renal failure only, heart failure only and combined renal and heart failure; Table S2: Anticoagulation therapy at admission to hospital and during the first days; Table S3: Reperfusion strategy during hospitalization.
