# Supplementary material for: Renal Failure and Systolic Heart Failure Have Synergistic Effect on In-Hospital All-Cause Mortality in Patients with Normotensive Acute Pulmonary Embolism
Source: Med Sci (Basel). 2025 Sep 10;13(3):183. doi: 10.3390/medsci13030183 (PMC12452358; doi:10.3390/medsci13030183)
Supplement: Supplementary file 1 [file medsci-13-00183-s001.zip › medsci-3842358-supplementary.pdf]

Figure S1. All-cause hospital death of acute, normotensive PE patients regarding the presence of renal failure using eGFR, heart failure or both (p<0.001).

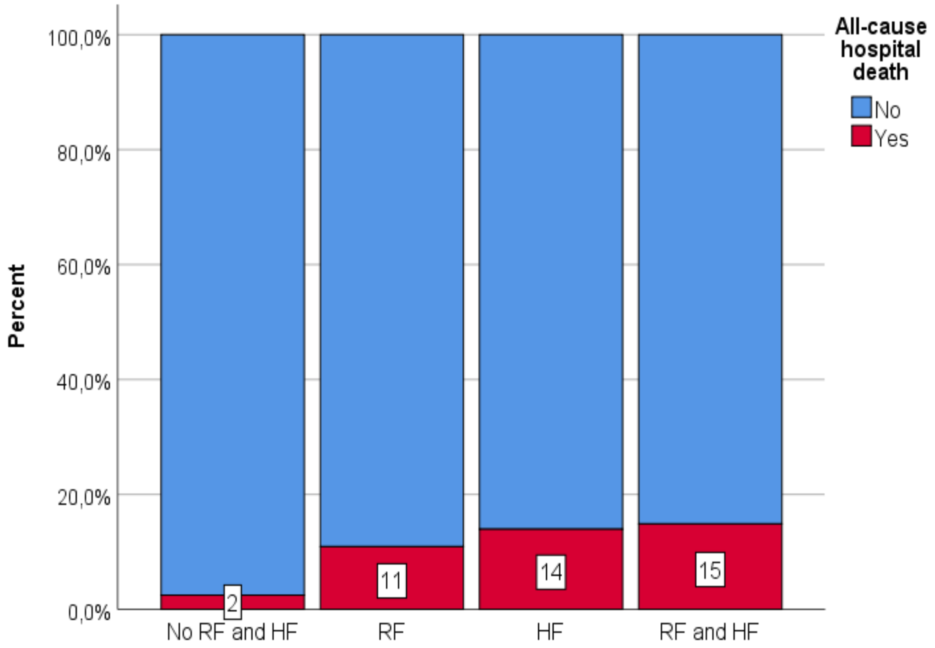

Table S1. All-cause death according the eGFR definition of renal failure (<60 ml/min/1.73m²) in patients without renal and heart failure, renal failure only, heart failure only and combined renal and heart failure.

Groups of patients	All-cause death	p
No RF or systolic HF	27 (2.5%)	<0.001
RF	73 (10.9%)	
sHF	12 (14.0)	
RF and sHF	18 (14.9%)	

Table S2. Anticoagulation therapy at admission to hospital and during the first days.

Anticoagulation therapy	Without renal and systolic heart failure N=1222	Renal failure N=503	Systolic heart failure N=96	Renal and systolic heart failure N=107	p
NFH – n (%)	428 (35.0)	160 (31.8)	19 (19.8)	20 (18.7)	0.001
LMWH – n (%)	762 (62.4)	331 (65.8)	76 (72.6)	81 (75.7)	
Fondaparinux – n (%)	13 (1.1)	5 (1.0)	0 (0.0)	4 (3.7)	
Oral anti-Xa – n (%)	19 (1.6)	7 (1.4)	1 (1.0)	2 (1.9)	

40 (2.0%) patients have not data about initial anticoagulation therapy.
NFH-nonfractional heparin;LMWH-low molecular weight heparin

Table S3. Reperfusion strategy during hospitalization.

Reperfusion strategy	Without renal and systolic heart failure N=1247	Renal failure N=514	Systolic heart failure N=99	Renal and systolic heart failure N=108	*p*
No reperfusion – *n* (%)	983 (78.8)	413 (80.4)	82 (82.8)	93 (86.1)	0.095
Classic tPA – *n* (%)	197 (15.8)	88 (17.1)	11 (11.1)	9 (8.3)	
Catheter directed thrombolysis – *n* (%)	62 (5.0)	15 (2.9)	5 (5.1)	5 (4.6)	
Mechanical catheter directed or surgical – *n* (%)	5 (0.4)	1 (0.2)	1 (1.0)	1 (0.9)	
